# Surface Evolution of Polymer Films Grown by Vapor Deposition: Growth of Local and Global Slopes of Interfaces

**DOI:** 10.3390/polym16111535

**Published:** 2024-05-29

**Authors:** Jungyu Shin, I. J. Lee

**Affiliations:** Department of Physics, Research Institute of Physics and Chemistry, Jeonbuk National University, Jeonju 54896, Republic of Korea

**Keywords:** surface roughness, kinetic roughening, polymer film growth, vapor deposition polymerization, atomic force microscopy

## Abstract

The kinetic roughening of polymer films grown by vapor deposition polymerization was analyzed using the widely accepted classification framework of “generic scaling ansatz” given for the structure factor. Over the past two decades, this method has played a pivotal role in classifying diverse forms of dynamic scaling and understanding the mechanisms driving interface roughening. The roughness exponents of the polymer films were consistently determined as $\alpha =1.25\pm 0.09,\;\alpha_{loc}=0.73\pm 0.02$, and $\alpha_{s}=0.99\pm 0.06$. However, the inability to unambiguously assign these roughness exponent values to a specific scaling subclass prompts the proposal of a practical alternative. This report illustrates how all potential dynamic scaling can be consistently identified and classified based on the relationship between two temporal scaling exponents measured in real space: the average local slope and the global slope of the interface. The intrinsic anomalous roughening class is conclusively assigned to polymer film growth characterized by anomalous “native (background slope-removed) local height fluctuations”. Moreover, the new analysis reveals that interfaces exhibiting anomalous scaling, previously classified as intrinsic anomalous roughening, could potentially belong to the super-rough class, particularly when the spectral roughness exponent $\alpha_{s}$ is equal to 1.

## 1. Introduction

Scale invariance, a phenomenon in which interface fluctuations exhibit some characteristic power laws in both time and space, is common in nature; growth fronts of solid films grown on flat surfaces, propagation of fire fronts, bacterial colony growth, and fluid flow in porous media are just a few examples [1,2]. The theory of kinetic roughening has provided a fundamental understanding of the growth mechanisms involved in modern thin film growth techniques, such as molecular beam epitaxy, chemical vapor deposition, and sputter deposition. A rough interface can be characterized by the global interface width, $W(L,t)={\langle {\left[h\left(x,t\right)-\langle h\left(x,t\right)\rangle \right]}^{2}\rangle }^{1/2}$, which is the root mean square fluctuation of surface height around its mean height value and the average $\langle \cdots \rangle$ is calculated over ***x*** in a system of size L. The kinetic roughening of an interface developing scale invariance of the correlation functions in space and time was understood using the Family–Vicsek (FV) dynamic scaling ansatz [1,2,3], $W(L,t)=t^{\beta }f(L/t^{1/z})$, wherein the scaling function f(u) behaves as $f(u)\sim u^{\alpha }$ if u≪1 and $f\left(u\right)\sim const$ if u≫1. The scaling exponents *β* and *α* characterize the growth of the roughening process and the roughness of the saturated interface, respectively. The interface width, at an early time, crosses into the saturation regime at a characteristic length called the lateral correlation length ξ. The dynamic exponent *z* quantifies the correlation length as $\xi (t)\sim t^{1/z}$ and links the exponents to the scaling law, z=α/β, which is valid for any growth process that follows the scaling ansatz. The fact that these scaling exponents do not depend on the microscopic details of the growth process has led to the concept of a universality class, in which the set of exponents is determined by the symmetry, the dimensionality, and the type of noise of the system under consideration.

The interesting idea of classifying all growths of rough interfaces into a few universality classes sharing the same set of scaling exponents determined by certain symmetries and conservation laws has not been successful. In practice, many growth systems exhibiting scale invariance (power law scaling) are not necessarily accounted for by unique dynamic roughening process specified by a few universality classes originally conceived. Minor differences in local growth rules, such as those seen in the limited mobility nonequilibrium growth models introduced by Wolf and Villain (WV) [4] and by Das Sarma and Tamborenea (DT) [5], lead to dramatic differences in growth morphology and universality class. The same growth rules, but a simple change in dimensionality, also results in different universality classes. The WV model described by the Edwards and Wilkinson (EW) [6] class in the (1 + 1)-dimension exhibits unstable mound growth in the (2 + 1)-dimension, whereas the DT model corresponding to nonlinear molecular beam epitaxy (MBE) growth in the (1 + 1)-dimension changes to a flat and very smooth EW universality class in the (2 + 1)-dimension [7]. Moreover, it has been shown that the scaling exponents can be continuously tuned by the growth parameters such as the disorder intensity in a random diffusion model [8,9] or the sticking coefficient in a re-emission model [10]. This argument against universality stems from the existence of a diverging mean local slope that goes into the scaling of local height fluctuations [11,12], which leads to a deviation from the normal FV scaling. Thus, the anomalous roughening of the interface refers to a growth system in which the global roughness exponent is different from the local ($\alpha \;\neq \;\alpha_{loc}$) [8,12]. The local roughness exponent $\alpha_{loc}$, which characterizes the scaling behavior of the local interface fluctuations or the local interface width at a fixed time, is practically determined by the height difference correlation function defined by $H\left(r\right)=\langle {\left|h\left(x+r\right)-h(x)\right|}^{2}\rangle =2W^{2}-2C(r)$. Here, $W=\sqrt{\langle {\left|h\left(x\right)-\langle h\rangle \right|}^{2}\rangle }$ is the root-mean-square roughness or saturation interface width, and $C\left(r\right)=\langle h\left(x+r\right)h(x)\rangle$ is the autocorrelation function, averaged over all ***x*** in windows of size r<L. It is easy to see that $H\left(r=\xi \right)\;\sim \;W^{2}$ for a window size equal to the correlation length. The height difference correlation function (or equivalent to the square of the local interface width, ${W\left(r,t\right)}^{2}$) behaves as [8,9,12].

(1) Hr,t ~ r2αloct2κloc            for r≪t1/z    t2β                       for r≫t1/z, 

where $\kappa_{loc}$ is the local slope exponent defined as $\kappa_{loc}=(\alpha -\alpha_{loc})/z$. The correlation length is given by $\xi (t)\sim t^{1/z}$ for $t^{1/z}\ll L$ and $\xi \left(t\right)=L$ for $t^{1/z}\gg L$. Therefore, if the standard FV scaling fails ($\alpha \neq \alpha_{loc}$), this scaling property qualifies $\alpha_{loc}$ as another independent roughness exponent that characterizes local interface fluctuations. There exists a wide range scaling exponents that deviate from the conventional universality classes due to contributions from diverging local slopes ($\kappa_{loc}>0$). Therefore, accurately identifying the different forms of dynamic scaling and classifying them into their respective scaling subclasses that share the same scaling properties is the first step to advance our understanding of roughening mechanism and thereby achieve the ultimate goal, namely surface roughness control.

The so-called generic dynamic scaling ansatz was proposed by Ramasco et al. [13] for the (2 + 1)-dimensional height structure factor or power spectrum as

(2) Sk,t ~ k−(2αs+2)t2(α−αs)/z               for kt1/z≫1  t(2α+2)/z                               for kt1/z≪1,   

in which the exponent $\alpha_{s}$ is the spectral roughness exponent. The (2 + 1)-dimension refers to a two-dimensional interface embedded in a three-dimensional space. The height difference correlation function $H\left(r,t\right)$ and the structure factor $S\left(k,t\right)$ are related by a Fourier transform [9,14].

(3) $$H\left(r,t\right)\propto \frac{1}{{\left(2\pi \right)}^{2}}\int_{2\pi /L}^{\pi /r_{0}}d^{2}k\left[1-\cos\left(kr\right)\right]S(k,t)$$

where $r_{0}$ is the lattice spacing. In the relationship of Equation (3), all possible types of dynamic scaling are defined by the value of the third independent exponent $\alpha_{s}$ and its relationship to $\alpha \;and\;\alpha_{loc}$, summarized as follows [13]:

(4) if αs<1→αloc=αs     αs=α→ Family-Vicsek αs≠α→ intrinsic   if αs>1→αloc=1         αs=α→ super-rough αs≠α→ new class.      

Since the correlation functions $H\left(r,t\right)$ and $S\left(k,t\right)$ are mathematically equivalent, as they are related by Fourier transform, the spectral roughness exponent $\alpha_{s}$ serves as a local roughness exponent generalized to take on all values including one. Unlike the value of $\alpha_{loc}$, which has a mathematical upper limit of 1 according to the definition of $H\left(r,t\right)$, the value of $\alpha_{s}$ can exceed one. This classification scheme, governed by these three roughness exponents, has been widely considered complete [15,16,17,18,19,20,21,22,23,24,25,26] and the interface roughening mechanism has been interpreted accordingly. In fact, various film growths by different techniques have been classified into subclasses of anomalous scaling: new class (or faceted surface) [17,20], intrinsic anomalous roughening [15,22,23,26], and super-rough interfaces [16,18,19,21]. However, as seen in some real-world film growth systems, the application of classification scheme based on the values of roughness exponents ($\alpha ,\;\alpha_{loc},\alpha_{s})$ is not always straightforward and often leads to inconclusive results for various reasons [15,16,17,18,19,20,21]. For example, the local roughness exponents determined from Equation (1) for r≪ξ (i.e., $H\left(r,t\right)\sim r^{{2\alpha }_{loc}}$) may be given incorrectly due to some artifacts, including finite-size effects [8,14]. Despite the widespread adoption of these approaches shown in Equation (4) as standard procedure for dynamic scaling analysis over the past two decades, we argue that this classification framework is impractical and incomplete. In this report, we present detailed experimental results observed in polymer films that support our claims. As an alternative, we propose a straightforward and comprehensive classification methodology based on the relationship between the average local and global slopes of interface, validated across a spectrum of growth systems, including polymer films.

## 2. Experimental Methods

**Figure 1:**
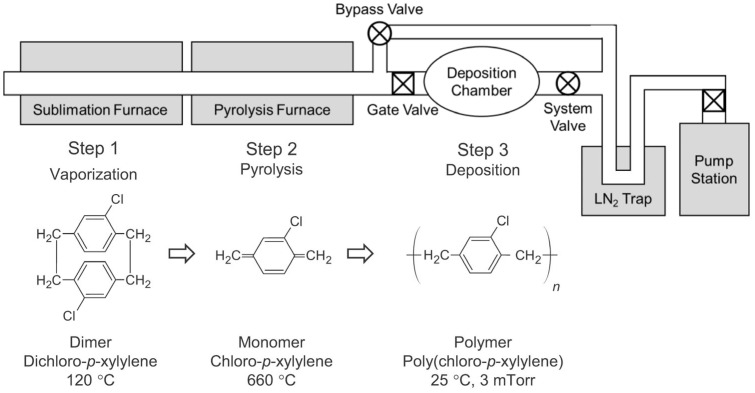
Schematic diagram of CVD setup consisting of a sublimation furnace, pyrolysis furnace, deposition chamber, and pump station. With the bypass valve closed, both the gate valve and the system valve are opened to initiate polymer film growth on the substrate.

Polymer films (parylene-C) were grown by conventional vapor deposition polymerization methods or the Gorham process [27] in custom chemical vapor deposition systems (CVD) [16,18]. A schematic diagram of the CVD setup is shown in Figure 1. Dimer molecules (dichloro-*p*-xylylene) (Daisan Kasei Co., Tokyo, Japan) were sublimated at 120 °C, dissociated into monomers in a pyrolysis furnace at 660 °C, and then polymerized on clean silicon substrates in a deposition chamber at room temperature. The CVD systems are equipped with a bypass valve that prevents monomers from entering the deposition chamber before the sublimation furnace has stabilized at the target temperature. By closing the bypass valve and opening the gate valve, a uniform monomer flux was exposed to the silicon substrate (Silicon Technology Co., Tokyo, Japan) in the deposition chamber. A near-constant pressure of ~3 mTorr was maintained during deposition, resulting in a film growth rate of ~30 nm/min in a steady growth regime for film thickness above ~200 nm. Substrates exposed to the monomer flux for the target time were taken from the deposition chamber and scanned in a noncontact mode using an atomic force microscopy (AFM) (XE100, Park Systems, Suwon, Republic of Korea). A non-contact high frequency AFM tip (PPP-NCHR, Nanosensors, Neuchatel, Switzerland) with a nominal resonance frequency of 330 kHz and a typical tip radius of less than 7 nm was used. Topographic AFM images of the parylene-C films were taken from at least four different locations on each polymer film with a scan size of 3 × 3 μm^2^ at a pixel resolution of 512 × 512. After removing thermal drift from the AFM images, basic surface analysis was performed with the factory installed analysis software (XEI, Ver. 4.3.4). The radially averaged correlation function was calculated based on the two-dimensional matrix converted from each AFM image. The film thickness on the silicon substrate was measured by spectroscopic ellipsometry (MG-1000UV, Nano-View, Ansan-si, Republic of Korea). Other details of the experimental methods are described in a recent study [28].

## 3. Results and Discussions

### 3.1. Kinetic Roughening of Parylene Films

**Figure 2:**
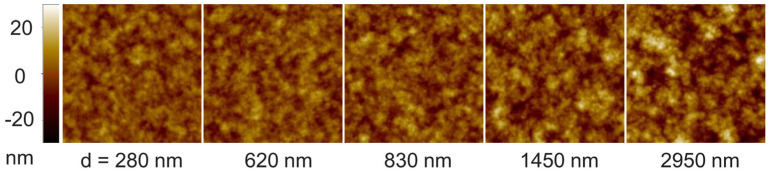
Representative AFM-scanned images of parylene-C films in the steady growth regime above film thickness d = 200 nm. A scan size of 3 × 3 μm^2^ with 512-pixel resolution is maintained for each measurement.

**Figure 3:**
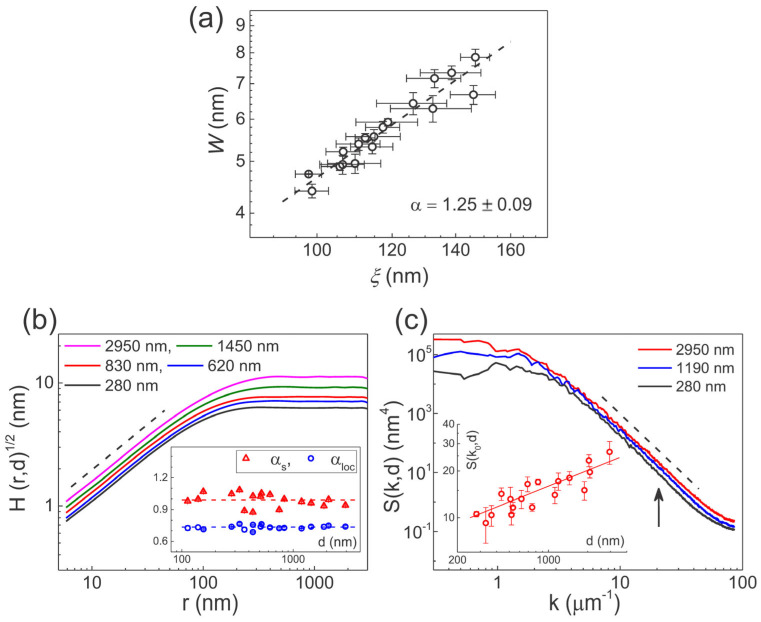
(**a**) Saturation interface width versus correlation length in a log–log plot. The dashed line represents a linear best fit giving α=1.25±0.09. (**b**) Logarithmic plot of height difference correlation function versus radial distance r at several representative film thicknesses. The dashed line, the guide for the eye, shows a linear slope of 0.73 corresponding to $\alpha_{loc}$. Inset displays the thickness dependence of the local ($\alpha_{loc}$) and spectral ($\alpha_{s}$) exponents in open circles and triangles, respectively. (**c**) Logarithmic plot of structure factor at several representative film thickness. The dashed line represents the linear slope of −4 corresponding to $-(2\alpha_{s}+2)$. Inset shows the log–log plot of the thickness dependence of $S(k_{0},d)$ at $k_{0}=20\;{\mu m}^{-1}$ indicated by the arrow. The error bars show the standard deviation from the average determined from at least four different locations on each film surface.

Representative AFM data from over 100 scans at 18 different film thicknesses from d= 100 nm to 3 μm are shown in Figure 2. To ensure reliable estimation of the scaling exponents, a sufficiently small pixel size ($r_{0}\ll \xi$) and sufficiently large scan size (r≫ξ) are maintained in all the AFM scan images. In finer scans, mound-like surface structures with round tops and sharp edge boundaries are found throughout the steady growth regime. The saturation interface width (W) and correlation length (ξ) increase as $W\sim d^{0.22\pm 0.01}\;and\;\xi \sim d^{0.17\pm 0.01}$ as the film thickness increases (see Supplementary Figure S1). The correlation length is determined from the radius-averaged auto correlation function C(r) using the relationship $C(\xi )/C(0)=e^{-1}$ for a given film thickness. Using the conventional scaling relation $W\sim {{(d}^{1/z})}^{\alpha }\sim \xi^{\alpha }$, the global roughness exponent is obtained as α=1.25±0.09, as displayed in Figure 3a. The range of the error bar represents the variation between at least four different measurements at each film thickness. Larger uncertainties are mainly associated with estimates of correlation lengths. The local roughness exponent $\alpha_{loc}$ and spectral roughness exponent $\alpha_{s}$ can be determined from the height difference correlation function and structure factor, respectively, which are shown in Figure 3b,c, for several representative film thicknesses. The radial correlation functions $H\left(r,t\right)$ and $S\left(k,t\right)$ exhibit scale invariance in both space and time (or film thickness) as defined by Equation (1) and Equation (2), respectively. A clear up-ward shift in ${H\left(r,d\right)}^{1/2}$ implies an anomalous scaling in which $\alpha \neq \alpha_{loc}$ (or $k_{loc}>0$). For a given film thickness, $\alpha_{loc}$ is determined from the best fit ${H\left(r\right)}^{1/2}\sim \;r^{\alpha_{loc}}$ in the range r≪ξ. As shown in the inset of panel (b), the local roughness exponents remain at the same value $\alpha_{loc}=0.73\pm 0.02$ in the steady growth regime, while the correlation length increases from 100 nm to 150 nm. Similarly, the thickness dependent $S\left(k,d\right)$ is resolved unambiguously, as shown in the inset of panel (c), where $S\left(k_{0},d\right)$ is shown as an example at $k_{0}=20\;\mu$m^−1^. These results are in contrast to previous report on growth of the same polymer film [16], where α was measured close to $\alpha_{s}$ (i.e., $\alpha \approx \alpha_{s}=0.93\pm 0.04$), as the thickness dependence of S(k,d) could not be resolved beyond the noise level. We will discuss this issue later. The value of $\alpha_{s}$ is determined from the *k*-dependence of S(k) in the high-*k* regime ($kt^{1/z}\gg 1$) at a given film thickness. The inset of panel (b) shows that $\alpha_{s}=0.99\pm 0.06$ fits well over the full range of film thicknesses within the steady growth regime. The discrepancy between the roughness exponents $\alpha_{loc}$ and $\alpha_{s}$ appears to be real because there are no crossovers at large length scales of S(k,d) and the value of $\alpha_{loc}$ remains the same even as the correlation length increases from 100 nm to 150 nm. Therefore, crossover and finite system size effects that may make the determination of $\alpha_{loc}$ from $H\left(r\right)$ unreliable [14], appear to be irrelevant for the current measurements. The three characteristic roughness exponents are accurately determined from the high-resolution data as α=1.25±0.09, $\alpha_{s}=0.99\pm 0.06,$ and $\alpha_{loc}=0.73\pm 0.02$.

The presence of a scaling function for the growth system and the consistency of the scaling analysis are confirmed as each correlation function collapses into a single curve (see Supplementary Figure S2). From the fact that $\alpha_{s}\approx 1$ and $\alpha_{s}\neq \alpha$, the proposed classification method defined by Equation (4) opens the possibility for a new class (faceted surface), as well as intrinsic anomalous roughening class. However, since neither $\alpha_{s}=\alpha_{loc}$ nor $\alpha_{loc}=1$, parylene-C film growth cannot be classified into the subclasses shown in Equation (4), derived from the scaling ansatz for the structure factor. A similar problem in implementing Equation (4) can be found in a previous report on parylene-C films treated with O_2_ plasma, which gives rise to bifractal reticular patterns composed of self-aligned protuberances and underlying structures [19]. Scaling exponents characterizing the underlying pit structure are obtained as $\alpha =-1,\beta =-0.48\pm 0.03,1/z=0.54\pm 0.06,\alpha_{s}=1$, and $\alpha_{loc}=0.95\pm 0.02$, all of which are measured directly from various correlation functions [19]. For $\alpha_{s}$ close to 1, blindly applying the proposed classification method (Equation (4)) allows two equally feasible scaling classes: intrinsic anomalous scaling and a new class, both of which satisfy the anomalous scaling condition $\alpha \neq \alpha_{s}$. FV scaling in real space (i.e., $\kappa_{loc}<0$), but anomalous scaling in Fourier space (i.e., $\alpha \neq \alpha_{s}$) gives more weight to the new (or faceted) class than to intrinsic anomalous class. However, the growth system does not display faceted surfaces with a constant slope known to be associated with the new class [13]. Therefore, when applying Equation (4), the anomalous roughening ($\alpha \neq \alpha_{s}$) of the plasma-etched polymer surface cannot be classified into a specific scaling subclass. Nevertheless, the standard FV scaling was discussed [19] based only on a set of standard scaling exponents given as close to the EW model with conservative noise (i.e., α=−1,β=−0.5,1/z=0.5 [1]). So far, we have presented experimental data on polymer films that reveal the fundamental limitations of existing classification methods. Apparently, this limitation stems from the fact that when $\alpha_{s}$ is given close to one, the classification has to rely on the value of the local roughness exponent measured from the relation ${H(r)}^{1/2}\sim r^{\alpha_{loc}}$ at a given film thickness. In Siegert’s simulation study, it was noted that the determination of the roughness exponent $\alpha_{loc}$ from the *r*-dependence of the height difference correlation function can be influenced by finite system size and discreteness effects [14]. This influence may cause roughness exponents that were originally close to or equal to one to be underestimated. Additionally, the mid-range roughness exponent may be overestimated due to the dilation effects due to tip-surface interaction during AFM measurements [29]. Instead of these roughness (spatial) exponents, we propose to use time exponents determined from the time-dependent scaling relations of the average local and global slopes, corresponding to the lateral dimensions of the lattice size and the correlation length, respectively.

### 3.2. Growth of Local and Global Slopes of the Interface

In their study of the effect of slope instability on anomalously roughened interfaces, Pang et al. introduced the concept of “local surface slope” such that $P\left(r,t\right)=\left({\tilde{h}}_{r}\left(x,t\right)-{\langle h\left(x,t\right)\rangle }_{r}\right)/\left(x-{\langle x\rangle }_{r}\right)$, where ${\tilde{h}}_{r}\left(x,t\right)$ is the straight line segment (or background slope line) measured on a flat substrate within a local widow size *r* at a given time [30]. The “native (background slope removed) local height fluctuation” is characterized by the “native local surface width” $W_{n}\left(r,t\right)={\langle {\langle {\left(h\left(x,t\right)-\tilde{h}\left(x,t\right)\right)}^{2}\rangle }_{r}\rangle }^{1/2}$, which is the average local height fluctuation relative to the slope line, ${\tilde{h}}_{r}\left(x,t\right)$, within a window of size *r*. Therefore, the local surface width, $W\left(r,t\right)$, and the native local surface width, $W_{n}\left(r,t\right)$, are related to the average local surface slope as [30].

(5) $$W\left(r,t\right)-W_{n}\left(r,t\right)\sim {\langle P^{2}\left(r,t\right)\rangle }^{\frac{1}{2}}.$$

Clearly, the average local surface slope ${\langle P^{2}(r,t)\rangle }^{1/2}$ is closely related to all kinds of surface slopes, such as the average local slope and the average global slope, which behave as $\sqrt{H\left(r_{0},t\right)}\sim t^{\kappa_{loc}}$ and $\sqrt{H\left(\xi ,t\right)}/\xi \sim \xi^{\alpha_{loc}-1}t^{\kappa_{loc}}=t^{(\alpha -1)/z}=t^{\kappa_{G}}$, respectively. The average local slope is essentially the average adjacent step height separated by the lattice constant $r_{0}$ (i.e., ${\langle \bar{{\left(\nabla h\right)}^{2}}\rangle }^{1/2})$ [11,12]. The value of the local slope exponent $\kappa_{loc}$ and its relation to the global slope exponent $\kappa_{G}$ are useful for defining the scaling behaviors of the native (background slope removed) local surface width. The anomalous scaling of $W\left(r,t\right)$ is due to the contribution of diverging local slope (see the case $\kappa_{loc}>0$ in Equation (1)). Therefore, if the average local slope behaves in the same way as the average global slope (i.e., $0<\kappa_{loc}=\kappa_{G}$), then the anomalous temporal dependence shared by $W\left(r,t\right)$ and ${\langle P^{2}(r,t)\rangle }^{1/2}$ does not contribute to $W_{n}\left(r,t\right)$. That is, the native local surface width $W_{n}\left(r,t\right)$ must not have an anomalous time dependence. A growth surface characterized by $W_{n}\left(r,t\right)$ exhibiting normal FV scaling (i.e., saturated in the long-time regime $t\gg r^{z}$, where the correlation length ($\xi \sim t^{1/z}$) is much greater than the window size *r*) is classified as super-rough class. On the other hand, when the average local slope scaling differs from the average global slope (i.e., $0<\kappa_{loc}\neq \kappa_{G}$), $W_{n}\left(r,t\right)$ should retain some type of anomalous scaling, such as $W\left(r,t\right)$. Therefore, surface growths with anomalous scaling in the native local surface width are naturally classified as intrinsic anomalous roughening class. We show through a simple argument based on Equation (5) that the existence of an anomalous time dependence on the native local surface width $W_{n}\left(r,t\right)$ can be determined by the relationship between $\kappa_{loc}$ and $\kappa_{G}$. This relationship allows us to distinguish between super-roughing class and intrinsic anomalous roughening class, regardless of the system dimension. Consistent results were found in one-dimensional calculations performed by Pang et al. [31]; when $\alpha_{loc}=1$, $W_{n}\left(r,t\right)$ exhibited normal FV scaling, while $\alpha_{loc}<1$, $W_{n}\left(r,t\right)$ exhibited the same type of anomalous scaling as $W\left(r,t\right)$. All types of dynamic scaling identified in real-space can be classified based on the time dependence of correlation functions using the relationship between the average local slope exponent $\kappa_{loc}$ and the average global slope exponent $\kappa_{G}$, which can be summarized as follows:

(6) if κloc≤0 → κloc=κG=0→ self-similar  otherwise→  Family-Vicsek   if κloc>0 → κloc=κG→ super-rough κloc≠κG→ intrinsic.      

**Figure 4:**
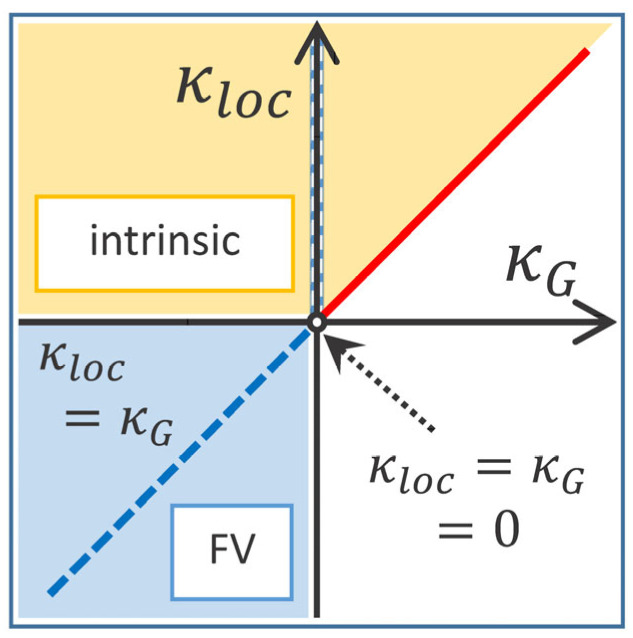
Types of dynamic scaling for growing interfaces mapped to the field of average local and global slope exponents. The condition that satisfies $\kappa_{loc}=\kappa_{G}$ is indicated by a diagonal line passing through the first and third quadrants. Slope selection of the interface occurs when $\kappa_{G}=0$, which is indicated by the hatched line satisfying $\kappa_{loc}>0$ within the intrinsic anomalous roughening class.

Conditions for all types of dynamic scaling are illustrated in Figure 4, in the field of average local and global slope exponents. Negative values of $\kappa_{loc}$ result in standard Family–Vicsek scaling ($i.e.,\alpha =\alpha_{loc})$, whereas diverging average local slopes ($\kappa_{loc}>0$) lead to anomalous scaling [11,12]. Isotropic or self-similar scaling occurs, by definition, at $\kappa_{loc}=\kappa_{G}=0$, where the global slope selection ($\kappa_{G}=0$) results in as a mounded or faceted surface in real space. From Figure 4, it is clear that mounded or faceted surfaces with slope selection also occur within the class of intrinsic anomalous roughening. When $\kappa_{loc}=\kappa_{G}$, conceivable normal FV-type scaling includes a self-similar class ($\kappa_{G}=0$) and an EW class ($\kappa_{G}<0$). Sneppen’s growth model-A, which produces a faceted surface with $\kappa_{G}=0$, was classified as a “new (or faceted) class” by Equation (4) [13] but is now classified as a self-similar class according to the new classification defined by Equation (6). Within this classification, two types of subclasses can be defined within the self-affine FV scaling, only if one considers a Flory-type scaling approach to compute local slope exponent in continuum growth models. One of subclasses belongs to the Kardar–Parisi–Zhang (KPZ) [32] class, whose characteristic exponents are determined by ${\;\kappa }_{loc}=-1/5$ and ${\;\kappa }_{G}=-1/3$ in the (1 + 1)-dimension [11], satisfying the condition${\;\kappa }_{G}\neq \kappa_{loc}\leq 0$. The other class is the EW class with equal global and local slope exponents of −1/4, satisfying the condition $\kappa_{G}=\kappa_{loc}<0$. For most surface roughening, the effect of a negative local slope exponents is transient and cannot be detected by the height difference correlation function. Nevertheless, it is an unexpected advantage that the FV class, defined in Equation (6), naturally includes smoothing processes where the average global slope exponent is measured as negative (α<0). Of course, classification Equation (6) and Equation (4) both define the same super-rough and intrinsic anomalous roughening classes, which can be easily seen by expressing both classification criteria as standard scaling exponents. The average local and global slope exponents are defined as $\kappa_{loc}=(\alpha -\alpha_{loc})/z$ and $\kappa_{G}=(\alpha -1)/z$, respectively. For the super-rough class, $\alpha >\alpha_{loc}=1$ in Equation (6) corresponds to $\alpha =\alpha_{s}>\alpha_{loc}=1$ in Equation (4). For the intrinsic class, $\alpha \neq \alpha_{loc}<1$ in Equation (6) corresponds to $\alpha \neq \alpha_{s}=\alpha_{loc}<1$ in Equation (4). The key reason is that anomalous scaling can be identified essentially based on whether $\alpha_{loc}$ is one or not, even without information about the structure factor ($\alpha_{s}$). Table 1 shows several case examples of continuum growth models where the standard and slope exponents can be calculated exactly. The set of scaling exponents of the random diffusion (RD) model is determined by a function of disorder parameter φ in the range 0<φ<1 and is classified into intrinsic anomalous roughening class [9]. The standard exponents of the conserved KPZ [33] (or Lai–Das Sarma–Villain (LDV)) model are given by $\alpha =\alpha_{loc}=2/3,\;z=10/3$ (or equivalently $\kappa_{loc}=0,\;\kappa_{G}=-1/10)$ in the (2 + 1)-dimension [34]. As the dimensionality increases, the scaling of the conserved KPZ equation changes from the intrinsic class ($\kappa_{G}\neq \kappa_{loc}>0$) to a KPZ-type FV scaling ($\kappa_{G}\neq \kappa_{loc}=0$), according to the new classification method. It is interesting to note that the relationship ($\kappa_{G}\neq \kappa_{loc}$) between the slope exponents is maintained even when the anomalous scaling disappears as the dimensionality increases. For the LMBE equation, the standard exponents are obtained analytically by $\alpha =\alpha_{loc}=1,\;z=4,\;\kappa_{loc}=0$ in (2 + 1)-dimension [34]. Similarly, as dimensionality increases, super-roughing ($\kappa_{G}=\kappa_{loc}>0$) changes to a self-similar class ($\kappa_{G}=\kappa_{loc}=0$), but the slope relation $\kappa_{G}=\kappa_{loc}$ is maintained. It seems to be reasonable to assume that the relationship between slope exponents is a fundamental property independent of the dimensionality of the growing system and is therefore a good candidate for a classification reference. However, we emphasize that the new classification method based on slope exponents is less practical when used in terms of a standard set of roughness exponents. This is mainly because $\alpha_{loc}$ is rarely measured as one, which is its mathematical upper limit. In fact, the values of $\alpha_{loc}$ measured between 0.78 and 0.90 are often taken as 1 for convenience in order to fit the subclasses defined by Equation (4) [16,17,18,19,20,21]. Moreover, as can be seen from the two representative experimental results discussed above, Equation (4) has the fundamental problem of having to determine whether $\alpha_{loc}=1$ and/or $\alpha_{loc}=\alpha_{s}$ when$\;\alpha_{s}$ is given close to 1. The new classification method shown in Equation (6), which is based on direct measurements from the time dependence of the average local and global slopes, provides a simple way to resolve the problem without invoking the scaling ansatz for the structure factor $S\left(k,t\right)$. In Table 1, it is interesting to note that the continuum local growth model LDV belongs to the intrinsic anomalous roughening class that satisfies $\kappa_{G}\neq \kappa_{loc}>0$, despite previous expectations for continuum growth models with local dynamics and spatiotemporal noise. Together with the simulation findings presented for the tensionless KPZ equation [26], the LDV model departs from the traditional assumptions regarding the role of nonlocal effects associated with the intrinsic anomalous roughening class of the interface. This challenges the conventional belief that intrinsic anomalous roughening is incompatible with local growth models [35].

### 3.3. Dynamic Scaling Classes of Parylene Film Growth

**Figure 5:**
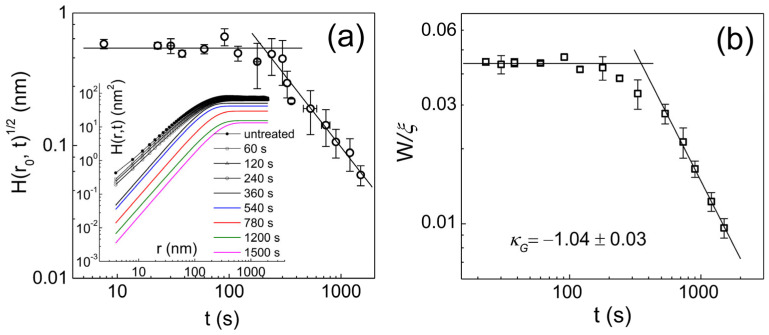
(**a**) The average adjacent step height $\sqrt{H(r_{0},t)}$ of underlying pit versus O_2_ plasma treatment time. The inset shows representative trace of $H\left(r\right)$ at various O_2_ plasma treatment times. The negative slope line corresponds to the local slope exponent $\kappa_{loc}=-1.08\pm 0.09$. (**b**) The average global slope W/ξ versus duration of O_2_ plasma treatment. A thin line with zero slope is a guide for the eye. A line with a negative slope is given by the least-square fit with an exponent $\kappa_{G}=-1.04\pm 0.03$. The error bars show the standard deviation from the mean value determined from at least four different locations on the surface of each film.

**Figure 6:**
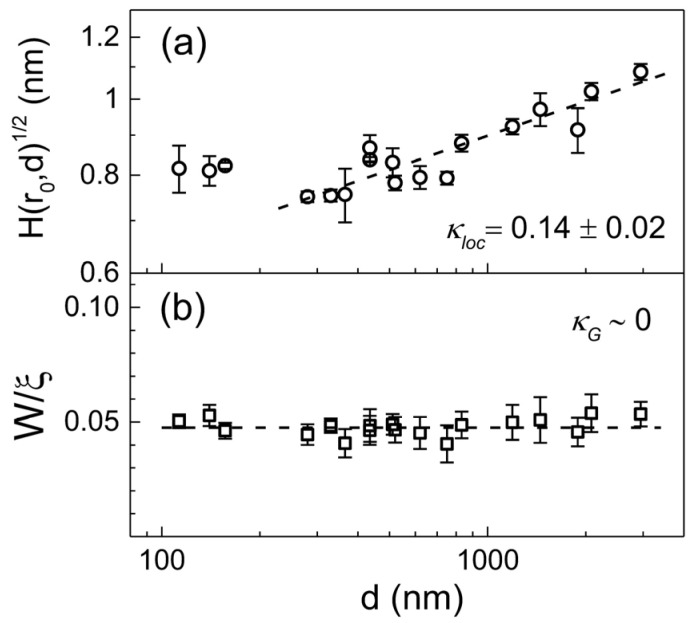
(**a**) The average local slope versus film thickness in the steady growth regime of parylene-C films above d~200 nm. The lattice spacing is given as $r_{0}=5.9$ nm. The dashed line is a guide for eye corresponding $\kappa_{loc}=0.14\pm 0.02$. (**b**) The averaged global slope (aspect ratio) of the interface versus the film thickness shows the formation of a slope-selected mound ($\kappa_{G}\sim 0$). The error bars show the standard deviation from the mean value determined from at least four different locations on the surface of each film.

We now show how to define a scaling subclass based on Equation (6) for the case discussed above where the existing classification method (Equation (4)) fails to define a scaling subclass. Representative traces of the H(r) function obtained from parylene-C films at various O_2_ plasma treatment times are displayed in the inset of Figure 5a. Data were constructed based on AFM images with a scan size of $2\times 2\;\mu m^{2}$ for each processing time, which is a new analysis based on the raw AFM data reported in Ref. [19]. As discussed earlier, the growth of the underlying pit structure above t~300 s cannot be specified, but some kind of anomalous scaling associated with $\alpha \neq \alpha_{s}$ is expected according to existing classification Equation (4). The temporal and spatial dependence of the correlation function H(r,t) exhibits clear changes with the generation of underlying pit structures. The stationary H(r) crossovers above t~300 s into the downward shifting H(r) with a steeper slope corresponding to $\alpha_{loc}=0.95\pm 0.02$. The average local slope $\sqrt{H\left(r_{0},t\right)}$ at various treatment times is shown in the main panel. The lattice spacing is given as $r_{0}=3.9$ nm. The thin line through the data points above t>200 s is a least- square fit of $t^{\kappa_{loc}}$ with $\kappa_{loc}=-1.08\pm 0.09.$ The average global slope ($\sqrt{H\left(\xi ,t\right)}/\xi \sim \;W/\xi$) shown in Figure 5b exhibits the power law scaling with an exponent of $\kappa_{G}=-1.04\pm 0.03$. According to the classification method (Equation (6)), the relation $\kappa_{loc}=\kappa_{G}<0$ extracted from the two plots indicates that the dynamic scaling belongs to self-affine FV scaling and is further subdivided into the EW type, which strongly supports the previous discussion based on the standard scaling exponents [19]. We point out that this polymer etching system is a textbook example showing that standard scaling exponents (α, β,1/z) can be interpreted into the respective local growth models, as long as the growth system is classified into the FV scaling class ($\kappa_{loc}\leq 0$). Naturally, the kinetic roughening of the underlying structure induced by O_2_ plasma can best be described as a smoothing process driven by surface tension, which redistributes irregularities of the interface. We now return to the pending problem of characterizing the kinetic roughening of parylene-C films grown on a silicon surface in a steady growth regime. The thickness (or temporal) dependence of the height difference correlation function at $r_{0}=5.9$ nm is shown in Figure 6a. A power law fit of the data gives the average local slope exponent with $\kappa_{loc}=0.14\pm 0.02$. Unlike the average local slope, Figure 6b indicates that the average global slope remains nearly constant. The formation of terminal topography associated with global slope selection behavior during vapor deposition polymerization is a separate issue that has been discussed very recently [28] and is therefore not discussed here. Based on Figure 3 and Figure 6, the characteristic scaling exponents of the parylene-C films are obtained as $\alpha =1.25\pm 0.10,\;\alpha_{s}=0.99\pm 0.06,\;\alpha_{loc}=0.73\pm 0.02,\;\kappa_{loc}=0.14\pm 0.02$, and $\kappa_{G}\sim 0$ in the steady growth regime above d~200 nm. We emphasize that the scaling properties of parylene-C film growth, which are neither $\alpha_{s}=\alpha_{loc}$ nor $\alpha_{loc}=1$, cannot be classified by Equation (4). However, within the new classification, the relationship $\kappa_{G}\neq \kappa_{loc}>0$, identified based on high-quality data, indicates that parylene-C film growth belongs to the intrinsic anomalous roughening class in which the native local surface width does not saturate over time in the long-time regime (i.e., $t\gg r^{z}$). The observation of mounds with a slope selection ($\kappa_{G}=0$) is one of the surface features that intrinsic anomalous roughening can provide (see Figure 4). Previously, scaling analysis of parylene-C films under steady growth regime reported the critical roughness exponents as $\alpha_{s}\approx \alpha =0.93\pm 0.04$ and $\alpha_{loc}=0.85\pm 0.03$ [16]. The relationship between the roughness exponents was considered to be $\alpha_{s}=\alpha \neq \alpha_{loc}$ and a super-rough class was claimed even though the measurements of $\alpha_{s}$ and $\alpha_{loc}$ were given much less than expected. The roughening mechanism was interpreted in association with fourth-order surface diffusion ($-K\nabla^{4}h$), despite some significant discrepancies in roughness exponent values [16]. However, slope analysis of the previous data, which displays the scaling relation $0<\kappa_{loc\;}(i.e.,\;\alpha >\alpha_{loc})\neq \kappa_{G\;}(\sim 0)$ [16,28], suggests an intrinsic anomalous roughening class that is consistent with the current data and analysis. Despite the discernible differences in the measured roughness exponents between the current and previous experiments, the new classification method allows consistent conclusion to be drawn. In addition, the standard scaling exponents of parylene-N, one of the parylene series, were measured as β=0.25±0.03, 1/z=0.31±0.02, $\alpha_{loc}=0.72\pm 0.05,\;and\;\kappa_{loc}=0.16\pm 0.02$, which was interpreted to indicate the nonlocal bulk diffusion [36]. Although the spectral roughness exponent $\alpha_{s}=0.5$ expected for the bulk diffusion mechanism is too small to apply to parylene-C or N, it is clear that the scaling subclasses belong to the same intrinsic anomalous roughening class satisfying $\kappa_{loc}\neq \kappa_{G}\approx 0$ [28]. The relationship based on time exponents $\kappa_{loc}$ and $\kappa_{G}$ appears to be robust, as evidenced by the dimensional analysis of the continuum growth equations in Table 1, and provide a consistent scaling analysis for parylene films regardless of parylene type and experimental details.

In contrast to this new classification method, the classification framework shown in Equation (4) is based on the mathematical equivalence between the height difference correlation function and the proposed generalized structure factor. The spectral roughness exponent $\alpha_{s}$ is a local roughness exponent defined in Fourier space generalized to take all values, including those greater than or equal to one. This new classification based on the slope exponent provides an interesting new perspective on the case where $\alpha_{s}=1$, which occurs in real growth systems but is not well defined by the current classification Equation (4). When $\alpha_{s}=1$ exactly, the growth system lies on the boundary between $\alpha_{s}>1$ and $\alpha_{s}<1$ and satisfies $\alpha_{s}=\alpha_{loc}=1$. Therefore, three distinct scaling classes are possible depending on the global roughness exponent α, but exclude the intrinsic anomalous class. If α=1, it implies $\kappa_{loc}=\kappa_{G}=0$ and belongs to a self-similar class, as can be seen in LMBE in the (2 + 1) dimension. If α<1, it means $\kappa_{loc}=\kappa_{G}<0$, which can be classified as EW type FV scaling, as seen in parylene-C etching system (i.e., EW with conserved noise). If α>1, it means $\kappa_{loc}=\kappa_{G}>0$, which belongs to the super-rough class where the native (background slope removed) local fluctuations behave according to standard FV scaling. This special case provides the unexpected insight that even in the super-rough class, both the correlation functions $H\left(r,t\right)$ and $S\left(k,t\right)$ can exhibit time dependence, which according to the existing classification, has been considered as a fingerprint of the intrinsic anomalous roughening class. Given that both $\alpha_{s}$ and $\alpha_{loc}$ are measured close to one, previous experimental results for TiN films [26], and (possibly) VO_2_ films [23] classified as intrinsic anomalous roughening class, may actually belong to the super-rough class. However, when it comes to the growth of parylene-C film, categorized within the intrinsic anomalous roughening class, it is imperative that the actual $\alpha_{s}$ value should be less than one, even though the consistently measured exponent value may appear equal to one (i.e., $\alpha_{s}=0.99\pm 0.06$).

Previous studies based on the dynamic renormalization group argument suggest that intrinsic anomalous roughening cannot occur in local growth models and requires nonlocal effects [35]. The usual origin of nonlocal effects involved in CVD growth are shadowing and re-emission [10]. The remitted particles can reach any part of the surface, thus nullifying the shadowing effect. A columnar-like morphology due to a severe shadowing effect at high sticking coefficient changes to a self-affine surface with a decreasing sticking coefficient. However, the parylene deposition process, which involves a very low sticking coefficient at the order of 10^−4^ at room temperature [37], while maintaining a non-self-affine surface, is inconsistent with the re-emission model. Parylene-N film growth was claimed for a nonlocal growth mechanism involving bulk diffusion [36]. However, the spectral roughness exponent $\alpha_{s}=0.5$ estimated for the bulk diffusion mechanism was too small to apply to parylene-C. Nevertheless, we have shown that the scaling subclasses of parylene-N and C films belong to the same intrinsic anomalous roughening. So far, typical nonlocal effects, such as shadowing and bulk diffusion, do not appear to be significantly relevant to parylene film growth. We also note that, contrary to long-held belief, local continuum growth models such as the one-dimensional conserved KPZ (or LDV) model appear to exhibit the intrinsic anomalous roughening. It seems reasonable to consider that nonlocal effects are not a necessary condition for a class of intrinsic anomalous roughening. Analysis based on the new classification method appears to indicate that the conserved dynamics involving the lateral growth term ${\left(\nabla h\right)}^{2}$ (LDV model) is associated with anomalous native (background slope removed) local height fluctuations, resulting in an intrinsic anomalous roughening of the interface. Nevertheless, for parylene film growth, which occurs physically in the (2 + 1) dimension, local growth models may not be sufficient to account for the intrinsic anomalous roughening. Parylene film growth occurs as monomer molecules attach either to the active ends of an existing polymer chain (propagation) or to other free monomers (initiation) [37,38]. This growth process is governed by a chemical reaction-limited aggregation mechanism where, unlike typical inorganic film growth, the interplay between diffusion and deposition (or flux) does not determine the typical timeframe of film growth [18,38]. The chemical nature of linear chain polymer growth can limit the number and distribution of available bonding sites [28], from which impinging monomer molecules may break randomness and develop preferential pathways before deposition on the surface, resulting in a kind of nonlocal effect.

## 4. Conclusions

We have investigated the surface evolution of polymer (parylene-C) film during a steady growth regime by analyzing data from over 100 scans across 18 film thicknesses. This study challenges the commonly accepted dynamic scaling theory used to identify and classify scaling subclasses. Despite its status as a comprehensive framework over the past two decades, uncertainty persists in accurately determining these scaling subclasses within current generic scaling ansatz approaches. This is especially true in cases where local roughness exponent values are measured close to one, such as parylene film growth and etching, and TiN and VO_2_ film growth. In response to these challenges, we propose an alternative classification method that relies on the relationship between two temporal scaling exponents in real space: the average local slope and the global slope of the interface. This new approach is demonstrated to accurately and consistently identify super-rough class and intrinsic anomalous class across a variety of continuum growth models and polymer film growth/etching systems without relying on the generic scaling ansatz given for the structure factor. Additionally, a detailed comparative analysis between the existing and new classifications shows the surprising result that the super-rough class can contain non-FV scaling properties in both real and Fourier spaces. This finding is quite unexpected, as within existing classification frameworks, this non-FV scaling behavior has often been interpreted as the fingerprint of an intrinsic anomalous roughening class. The surface evolution during parylene film growth and O_2_ plasma etching is unambiguously and consistently classified into intrinsic anomalous roughening and EW type self-affine FV classes, respectively. These findings suggest that polymer film growth leads to anomalous native local height fluctuations, while polymer etching is dominated by a surface tension-driven smoothing process, resulting in a self-affine surface. The recognition and identification of native local height fluctuations as a fundamental issue in anomalous kinetic roughening of interfaces underscores the necessity for further research in this area.

## Figures and Tables

**Table 1:** Summary of standard and slope exponents for linear MBE (LMBE) [12], random diffusion (RD) [9], and Lai–Das Sarma–Villain (LDV) equations [33] in (1 + 1)-dimension.

Model	Equation	α	$\alpha_{loc}$	*z*	$\kappa_{loc}$	$\kappa_{G}$	Subclass
LMBE	$\partial h/\partial t=-K\nabla^{4}h$ +η(x,t)	$\frac{3}{2}$	1	4	$\frac{1}{8}$	$\frac{1}{8}$	super-rough [9,11,12]
RD	$\partial h/\partial t=\frac{\partial }{\partial x}\left[D\left(x\right)\frac{\partial h}{\partial x}\right]$ +η(x,t)	$\frac{1}{2-2\varphi }$	$\frac{1}{2}$	$\frac{2-\varphi }{1-\varphi }$	$\frac{\varphi }{4-2\varphi }$	$\frac{2\varphi -1}{4-2\varphi }$	intrinsic [9]
LDV	$\partial h/\partial t=-K\nabla^{4}h$ $+{\lambda \nabla }^{2}{\left(\nabla h\right)}^{2}+\eta (x,t)$	1	$\frac{8}{11}$	3	$\frac{1}{11}$	0	intrinsic

## Data Availability

Data are contained within the article and Supplementary Materials. Correspondence and requests for materials should be addressed to I. J. Lee.

## Supplementary Materials

The following supporting information can be downloaded at: https://www.mdpi.com/article/10.3390/polym16111535/s1, Figure S1. Saturated interface width (*W*) and correlation length (*ξ*) versus film thickness. Figure S2. Data collapse of the correlation functions shown in Figure 3.
