# Supplementary material for: Surface Evolution of Polymer Films Grown by Vapor Deposition: Growth of Local and Global Slopes of Interfaces
Source: Polymers (Basel). 2024 May 29;16(11):1535. doi: 10.3390/polym16111535 (PMC11175125; doi:10.3390/polym16111535)
Supplement: Supplementary file 1 [file polymers-16-01535-s001.zip › polymers-3012198-supplementary.pdf]

Supplementary Information

Surface Evolution of Polymer Films Grown by Vapor Deposition: Growth of Local and Global Slopes of Interfaces

*Jungyu Shin and I. J. Lee**

Department of Physics, Research Institute of Physics and Chemistry, Jeonbuk National University, Jeonju, 54896, Republic of Korea

* Correspondence and requests for materials should be addressed to I.J.L. (ijlee@jbnu.ac.kr)

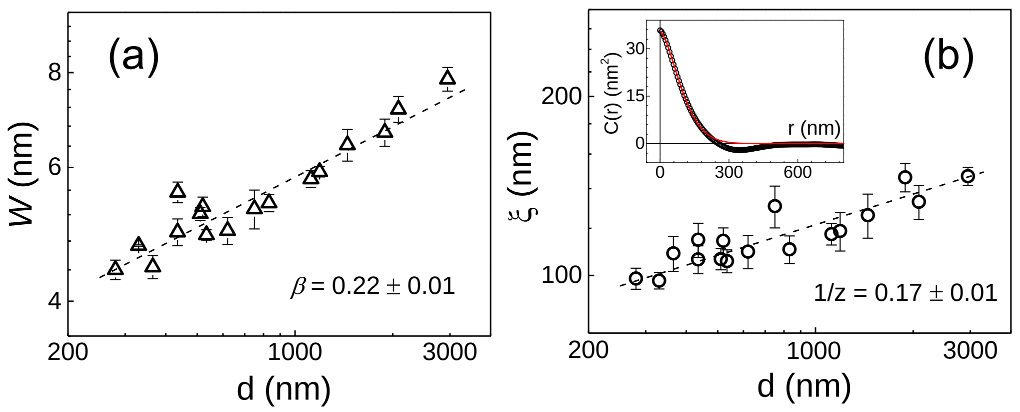

Figure S1. Saturated interface width (W) and correlation length (ξ) versus film thickness are shown in panel (a) and (b), respectively. The growth exponent (β) and dynamic exponent ($1/z$) are obtained as 0.22 ± 0.01 and 0.17 ± 0.01, respectively. The global roughness exponent determined from the scaling law $\alpha = z\beta = 1.29 \pm 0.10$ is consistent with the value obtained in Figure 3 in the main text. The inset of panel (b) shows that the correlation length is determined from the relation $C(\xi)/C(0) = \mathrm{e}^{-1}$ for a given film thickness.

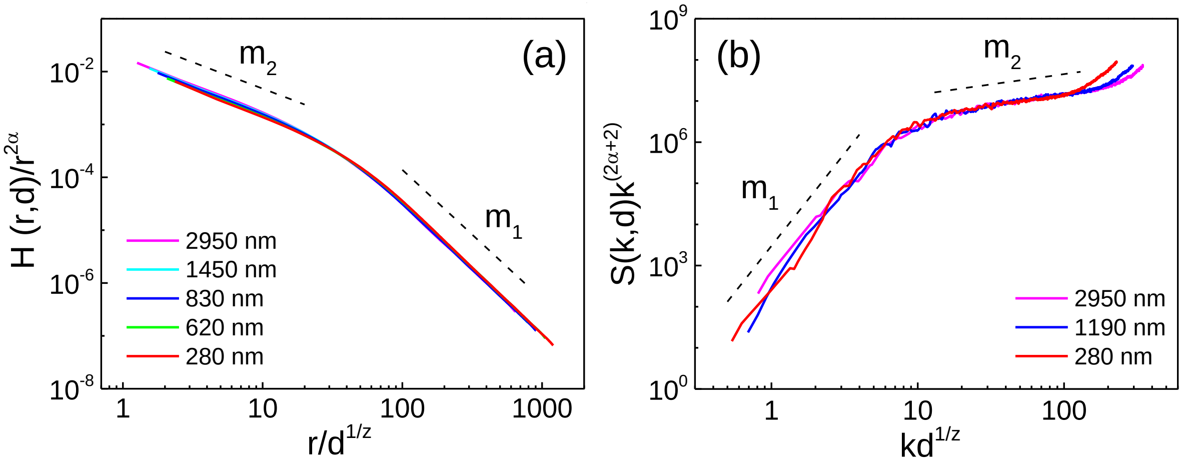

Figure S2. Data collapse of the correlation functions shown in Figure 3. Predetermined roughness exponent values with standard deviation $\alpha = 1.25 \pm 0.09$ and $1/z = 0.17 \pm 0.01$ are used for data collapse. High-quality data collapse indicates the presence of a scaling function $f(r/d^{1/z})$ even if the local scaling properties significantly differs from the global properties. Guide lines marked as $m_1 = -2.5$ and $m_2 = -1.0$ in panel (a) corresponds to -2α and $-2(\alpha - \alpha_{loc})$. The difference between the two slopes $-(m_1 - m_2)$ gives $\alpha_{loc} = 0.75$, which should be equal to the slope measured directly from $\sqrt{H(r,d)}$ in the small-*r* region ($r \ll \xi$(or $d^{1/z}$)), as shown in Figure 3(b). Panel (b) shows data collapse of the two-dimensional structure factors obtained for various film thicknesses. The slopes $m_1 = 4.5$ and $m_2 = 0.5$ correspond to $2\alpha + 2$ and $2(\alpha - \alpha_s)$ respectively. The difference $(m_1 - m_2) = 4$ gives $\alpha_s = 1$, which should be equal to the slope of $S(k,d)$ measured directly in the high-*k* regime ($k \gg \xi^{-1}$(or $d^{-1/z}$)), i.e. $\alpha_s = 0.99 \pm 0.06$ as determined in Figure 3(c).
